# Supplementary material for: Preparation and Characterization of Degradable Cellulose−Based Paper with Superhydrophobic, Antibacterial, and Barrier Properties for Food Packaging
Source: Int J Mol Sci. 2022 Sep 22;23(19):11158. doi: 10.3390/ijms231911158 (PMC9570331; doi:10.3390/ijms231911158)
Supplement: Supplementary file 1 [file ijms-23-11158-s001.zip › Supplementary Materials.pdf]

Supplementary Materials for

Preparation and Characterization of Degradable Cellulose–Based Paper with Superhydrophobic, Antibacterial and Barrier Properties for Food Packaging

Xiaofan Jiang [1], Qiang Li [1], Xintin Li [1], Yao Meng [1], Zhe Ling [2], Zhe Ji [1,3,*] and Fushan Chen [1,*]

[1] College of Marine Science and Bioengineering, Qingdao University of Science and Technology, Qingdao 266042, China; jxiaoff0330@163.com (X.J.); lqiang0627@163.com (Q.L.); lay812913@163.com (X.L.); mengyao@qust.edu.cn (Y.M.)

[2] Jiangsu Co–Innovation Center of Efficient Processing and Utilization of Forest Resources, College of Chemical Engineering, Nanjing Forestry University, Nanjing 210037, China; jjling19@njfu.edu.cn

[3] State Key Laboratory of Biobased Material and Green Papermaking, Qilu University of Technology, Shandong Academy of Sciences, Jinan 250353, China

* Correspondence: jizhe@qust.edu.cn (Z.J.); chenfushan@qust.edu.cn (F.C.)

Table S1. Tensile strength of samples.

Sample	CP	PC	PC-SA	PC-SA/SiO_2
TS/MPa	70.5±2	118.9±2	101.4±2	104.3±2

Table S2. Cobb of samples at different times.

Time	60s	300s	600s	1800s
CP	12.1±1.0	14.5±1.0	15.7±1.0	19.4±1.0
PC	0	0.7±1.0	6.7±1.0	17.3±1.0
PC-SA	0	0.6±1.0	4.5±1.0	15.5±1.0
PC-SA/SiO2	0	0	1.2±1.0	9.6±1.0

Table S3. Number of colonies in the samples.

Sample	CP	PLA	PC	PC-SA	PC-SA/SiO_2
E. coli	＞10^6	422±5	34±2	3±1	0
S. aureus	＞10^6	213±5	6±2	1±1	0
